# Molecular Origin of Polyglutamine Aggregation in Neurodegenerative Diseases 

**DOI:** 10.1371/journal.pcbi.0010030

**Published:** 2005-08-26

**Authors:** Sagar D Khare, Feng Ding, Kenneth N Gwanmesia, Nikolay V Dokholyan

**Affiliations:** 1 Department of Biochemistry and Biophysics, University of North Carolina, Chapel Hill, North Carolina, United States of America; 2 Department of Physics and Pre-Engineering, Delaware State University, Dover, Delaware, United States of America; Peking University, China

## Abstract

Expansion of polyglutamine (polyQ) tracts in proteins results in protein aggregation and is associated with cell death in at least nine neurodegenerative diseases. Disease age of onset is correlated with the polyQ insert length above a critical value of 35–40 glutamines. The aggregation kinetics of isolated polyQ peptides in vitro also shows a similar critical-length dependence. While recent experimental work has provided considerable insights into polyQ aggregation, the molecular mechanism of aggregation is not well understood. Here, using computer simulations of isolated polyQ peptides, we show that a mechanism of aggregation is the conformational transition in a single polyQ peptide chain from random coil to a parallel β-helix. This transition occurs selectively in peptides longer than 37 glutamines. In the β-helices observed in simulations, all residues adopt β-strand backbone dihedral angles, and the polypeptide chain coils around a central helical axis with 18.5 ± 2 residues per turn. We also find that mutant polyQ peptides with proline-glycine inserts show formation of antiparallel β-hairpins in their ground state, in agreement with experiments. The lower stability of mutant β-helices explains their lower aggregation rates compared to wild type. Our results provide a molecular mechanism for polyQ-mediated aggregation.

## Synopsis

Nine human diseases, including Huntington's disease, are associated with an expanded trinucleotide sequence CAG in genes. Since CAG codes for the amino acid glutamine, these disorders are collectively known as polyglutamine diseases. Although the genes (and proteins) involved in different polyglutamine diseases have little in common, the disorders they cause follow a strikingly similar course: If the length of the expansion exceeds a critical value of 35–40, the greater the number of glutamine repeats in a protein, the earlier the onset of disease and the more severe the symptoms. This fact suggests that abnormally long glutamine tracts render their host protein toxic to nerve cells, and all polyglutamine diseases are hypothesized to progress via common molecular mechanisms. One possible mechanism of cell death is that the abnormally long sequence of glutamines acquires a shape that prevents the host protein from folding into its proper shape. What is the structure acquired by polyglutamine and what is the molecular basis of the observed threshold repeat length? Using computer models of polyglutamine, the authors show that if, and only if, the length of polyglutamine repeats is longer than the critical value found in disease, it acquires a specific shape called a β-helix. The longer the glutamine tract length, the higher is the propensity to form β-helices. This length-dependent formation of β-helices by polyglutamine stretches may provide a unified molecular framework for understanding the structural basis of different trinucleotide repeat-associated diseases.

## Introduction

The appearance of polyglutamine (polyQ)-containing aggregates [1–3] is a hallmark of disease progression in all diseases in which CAG-expansions occur in genes [2]. Intranuclear inclusion bodies containing polyQ aggregates have been found in vitro [4,5], in cell cultures, animal models, and affected patients [6,7]. The aggregates are known to have a characteristic amyloid topology [8]. The inhibition of oligomerization by the azo-dye Congo red, or by the Hsp70/Hsp40 chaperone system, exerts marked protective effects in vivo and in vitro [9,10]. Aggregation and disease are observed if the number of glutamines in the expansion, *n,* exceeds a critical value, *n*_C_ (i.e., *n*_C_ = 35–40) [3]. The nearly universal existence of this criticality in all polyQ-related diseases (except in spinocerebellar ataxia 6) suggests that when the polyQ insert length exceeds a critical value (*n* > *n*_C_), a pathological change, largely independent of the host protein, occurs in the polyQ insert itself. Therefore, isolated polyQ peptides *(Q_n_)* have been used as model systems for studying polyQ aggregation [4,11,12], and it is known that: (a) The nuclear uptake of polyQ peptide aggregates prepared in vitro is cytotoxic in cell cultures [13], (b) isolated polyQ peptides have in vitro aggregation properties similar to the corresponding full-length proteins containing the polyQ insert [4,14], (c) peptide aggregation follows a nucleated mechanism showing characteristic lag and growth phases [5,11], and (d) the glutamine tract-length dependence of the lag-time interval correlates well with the age of onset of disease [11]. Peptides of subcritical lengths (*n* < 35–40) have long lag times of aggregation and a corresponding (predicted) age of onset later than the typical life span of a person. Longer peptides (*n* > 35–40) have progressively smaller lag times of aggregation, and a correspondingly early age of onset of the disease [11].

Unaggregated polyQ peptides form random coil structures, whereas aggregates are composed of amyloid-like β-strands [15]. The conversion of random coil to β-strand occurs in an individual polyQ chain [11], and fibril formation occurs by addition of other polyQ chains to these monomeric β-strand nuclei. Therefore, the conformational dynamics of an individual polyQ chain determines both its aggregation mechanism and the structure of the final aggregates. The details of the conformational dynamics of polyQ and the length dependence of the dynamics are not well understood [6].

## Results/Discussion

To elucidate the structural dynamics of single polyQ chains, we performed molecular dynamics (MD) simulations of simplified models of polyQ. An atomic-level representation of polyQ limits sampling efficiency in simulations, making it unsuitable to study the dynamics of aggregation. Therefore, we used a simplified pseudo-atom representation to capture the relevant degrees of freedom for aggregation. We introduced three types of nonbonded interactions between the glutamine pseudo-atoms: hydrophobic interactions between the glutamine methylene groups, geometrically determined hydrogen bonds between backbone NH and O atoms, and sidechain-backbone polar interactions between the sidechain carboxylamine group and the backbone NH or O atoms. Protein-solvent interactions play an important role in protein folding and aggregation. However, in simplified models of protein folding and aggregation the solvent interactions are considered implicitly [16]. In the interaction models employed in our study, the solvent effects were captured by the effective hydrophobic interactions between the methylene groups in the sidechain. We used the discrete molecular dynamics (DMD) algorithm [17] to study polyQ dynamics.

**Figure 1:**
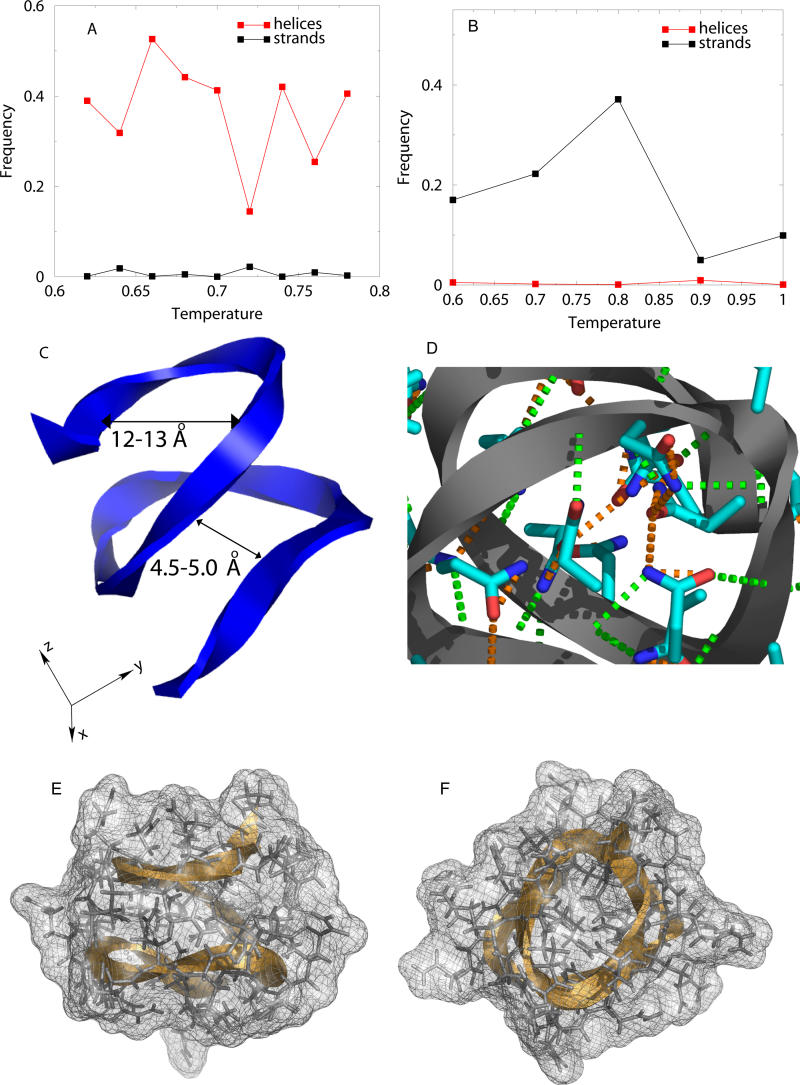
Dynamics of PolyQ Peptides (A, B) The fraction of secondary structure as a function of temperature for model polyQ peptides (A) without sidechain-backbone interactions, and (B) with sidechain-backbone interactions. Only helices and β-strands are shown; at each temperature the remainder is random coil. (C) A β-helix formed by a *Q*_37_ at the temperature *T* = 0.74. The width of the helix is 12–13 Å, and the distance between strands is 4–5 Å. (D) The network of possible sidechain-backbone (green) and sidechain-sidechain (orange) hydrogen bonding interactions in the core of the β-helix. Hydrogen bonds are shown as dashed lines, and bifurcations indicate the multiple possibilities for interaction partners. (E, F) Side (E) and top (F) views of the β-helix structure showing the well-packed core and outward-pointing sidechains.

The first question that we address is the following: What is the underlying minimal set of interactions responsible for the experimentally observed conformational transitions in polyQ aggregation? Since the conformational transition from random-coil polyQ to β-strand is known to be a nucleated process [11], we expect that an energy barrier is crossed during β-strand formation. Barrier crossing is enhanced as the system temperature is increased. Therefore, we study the dynamics of model polyQ peptides as a function of temperature. We hypothesized that the physical basis of the conformational change from random coil to β-strands is the presence of unique sidechain-backbone hydrogen bonding interactions in polyQ. To test this hypothesis, we performed simulations of a 37-mer (*Q*_37_) polyQ peptide with and without sidechain-backbone hydrogen bonding interactions. It is known that homopolymeric peptides with no sidechain-backbone interactions, e.g., polyalanine, form α-helices in their ground state [18,19], and at higher temperatures the helices melt to form a random coil [20] that then aggregates into a β-rich structure. We found that in the absence of sidechain-backbone interactions, polyQ dynamics are similar to polyalanine: It forms α-helices at low temperature (Figure 1A), and a random coil as the temperature is increased. A monomer peptide in this polyQ model does not form β-strands. In contrast, when sidechain-backbone hydrogen bonding is present, polyQ is a random coil at low temperatures, adopts a β-strand conformation in an intermediate range of temperatures, and is again a random coil at higher temperatures (Figure 1B).Thus, sidechain-backbone interactions lead to the formation of β-strands by a single polyQ peptide, which is the nucleating structural transition observed in polyQ-peptide aggregation.

**Figure 2:**
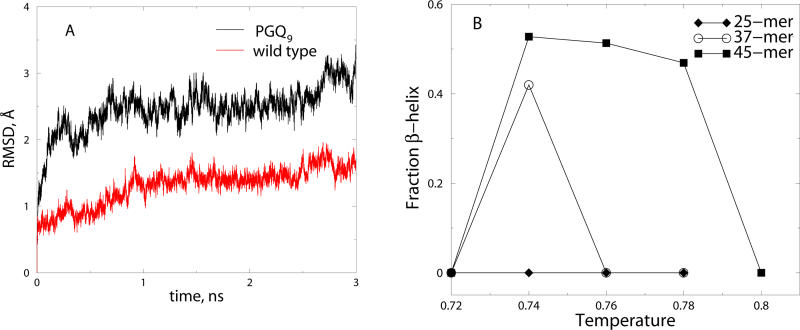
Formation and Stability of β-Helices (A) RMSD versus time in all-atom MD simulations of polyQ peptide β-helices for wild-type β-helix (red) and PG-Q_9_ β-helix (black). The PG-Q_9_ β-helix was constructed by replacing six glutamines in *Q*_37_ with the proline-glycine sequence inserted after every nine glutamines. (B) The fraction of β-helix content as a function of temperature for a 25-mer (diamonds), 37-mer (unfilled circles), and 45-mer (squares). The fraction of β-helix content was calculated by measuring the frequency of contacts between a residue *i* and residues *i* + 16 to *i* + 20, for all *i*.

Strikingly, the conformation adopted by a *Q*_37_ chain under conditions in which it adopts β-strand topologies (*T* = 0.72 to *T* = 0.78, in units of ɛ/*k*_B_, where ɛ is the energy unit and *k*_B_ is the Boltzmann's constant) is a parallel β-helix (Figure 1C). In these β-helices, all residues adopt β-strand backbone dihedral angles, and the polypeptide chain coils around a central helical axis. Several examples of such parallel β-helices (reviewed by Wetzel [21]) are found in the Protein Databank (http://www.rcsb.org/pdb/). For amyloid fibrils formed by polyQ, Perutz previously proposed a β-helix model based on X-ray diffraction data [8]. However, in contrast with Perutz's model, which has a central aqueous pore, the β-helices observed in our simulations are well packed, exclude the solvent, and are stabilized by buried sidechain-backbone and sidechain-sidechain hydrogen bonds (Figure 1D–1F). To evaluate whether these β-helix structures, once formed, have residence times long enough to propagate further aggregation, and to obtain better-defined thermodynamic ensembles, we evaluated the stability of β-helices at 300 Kelvin using all-atom MD simulations. As shown in Figure 2A, the polyQ structure remains stable on the nanosecond time scale accessible in all-atom simulations. If the formation of β-helices corresponds to the nucleation step in the aggregation reaction [5], and, assuming that the further elongation of the aggregate is diffusion-limited, the average time between protein collisions at a concentration of 100 μM is expected to be about 10 ns. Therefore, the observed stability of the polyQ β-helix on the nanosecond time scale is expected to be sufficient for further propagation of the aggregate.

Apart from sidechain-backbone interactions, a number of sidechain-sidechain interactions persisted throughout the MD simulation. We did not use sidechain-sidechain hydrogen bonding interactions in the DMD simulations used to generate β-helices. Thus, even though sidechain-sidechain interactions were not required for the formation of β-helices, they were nevertheless formed in β-helices. Further, these interactions were persistent throughout the length of the all-atom MD simulations, suggesting that they do play a significant role in the stabilization of the β-helices.

By characterizing the length dependence of β-helix formation, we uncovered the molecular basis of the observed glutamine length dependence of polyQ aggregation. Since the average number of residues per turn of the β-helix in our simulations is 18.5 ± 2 residues, we expected that about 33–40 glutamines would be required for its formation. To test this hypothesized length dependence of β-helix formation, we studied the conformational dynamics of 25-mer and 45-mer polyQ peptides and found that β-helices are absent at all temperatures when the repeat length was 25. Moreover, the β-helix topology was stable in a broader range of temperatures for the 45-mer (Figure 2B), demonstrating that β-helix formation increases with the length of polyQ. The formation of a β-helix from a random coil was accompanied by entropy loss, leading to a free energy barrier. This barrier results in the lag times observed in experiments of polyQ peptide aggregation [11]. Barrier crossing is enhanced for longer peptides, because the enthalpy gain upon β-helix formation compensates for the entropy loss in the transition. Therefore, β-helices are formed only by peptides longer than a critical length (*n* > *n*_C_). Recently, Stork et al. [22] found that the dimerization of two *Q*_37_ β-helices resulted in a stabilization of the (preformed, in their study) β-helix conformation. The stabilization of the β-helix upon dimerization shows that the dimerization is a downhill process (i.e., there is no energy barrier) on the free-energy landscape. Thus, we propose that length-dependent β-helix formation may be the molecular origin of polyglutamine-mediated aggregation. We also propose that once a β-helix is formed by a monomer, the elongation of the aggregate may involve the conversion of other chains to β-helices induced by the β-helix nucleus. A fibril may be formed by stacking of multiple β-helices, as suggested by Stork et al. [22], and these fibrils may arrange further to form larger fibers.

**Figure 3:**
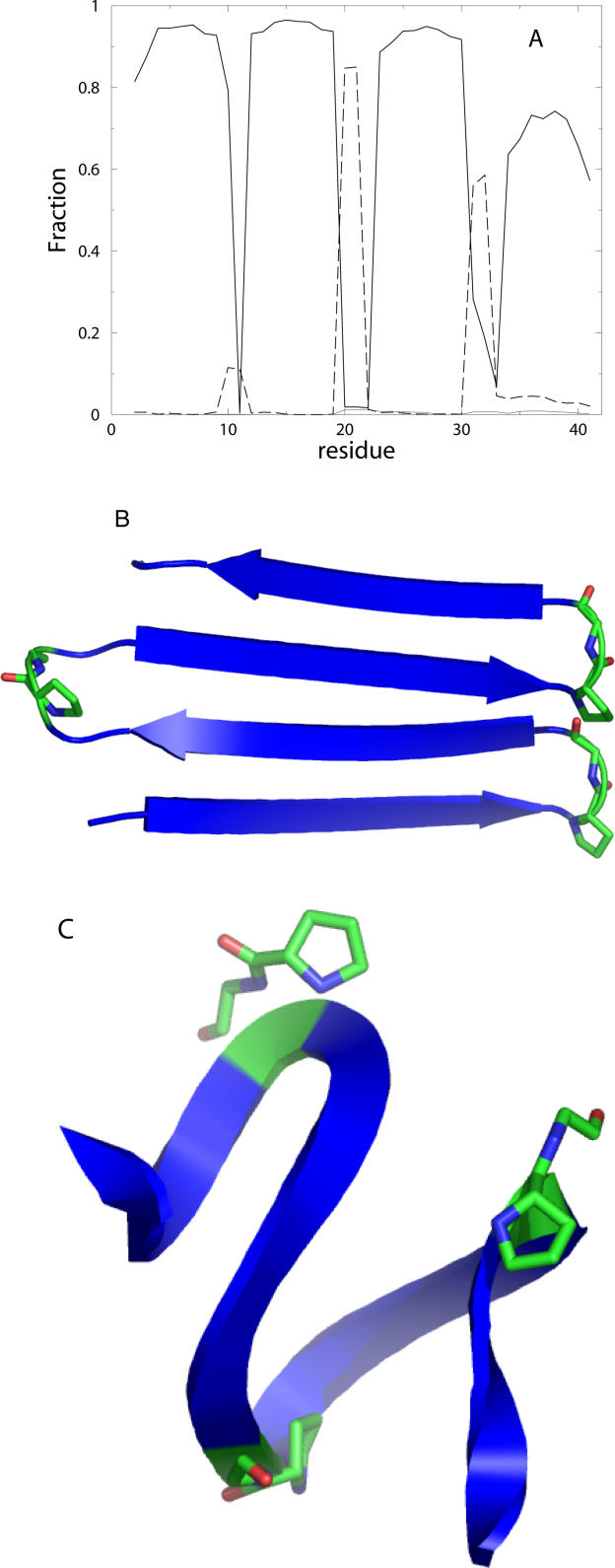
Characterization of PolyQ Mutants (A) Secondary structure formed by the PG-Q_9_ mutant at *T* = 0.74. The glutamines formed β-sheets (solid line) with high frequency, whereas the proline-glycine sequence formed turns (dashed line). (B) The β-hairpin formed by the PG-Q_9_ peptide in its ground state are shown; the proline and glycine residues are highlighted in green. (C) The β-helix conformation of PG-Q_9_ after 3 ns in MD simulations is shown; the turns in the β-helix (green) are formed at the proline-glycine insertion.

The formation of β-helices by a single polyQ chain can be used to rationalize the aggregation of experimentally characterized mutant polyQ peptides. Previously, Thakur and Wetzel [5] found that mutant polyQ peptides, in which the turn-inducing amino acid sequence proline-glycine was inserted at different sequence intervals—e.g., (Q_9_-PG-Q_9_)_3_ (PG-Q_9_) and (Q_10_-PG-Q_10_)_3_ (PG-Q_10_)—modulated the aggregation kinetics of polyQ peptides. The mutant PG-Q_9_ was found to aggregate at a marginally smaller rate than the wild-type polyQ, and the critical nucleus size for aggregation, as for the wild type, was one. The existence of nucleated aggregation kinetics of the mutant suggests that, similar to the wild type, barrier crossing from the ground state to an aggregation-prone state occurs. Therefore, we hypothesized that, similar to wild-type polyQ, a thermodynamically unfavorable nucleating conformational transition occurs in a single β-hairpin forming PGQ_9_ peptide. We studied the conformational dynamics of the PGQ_9_-mutant peptide and found that, in agreement with Thakur and Wetzel's prediction, this peptide forms a four-stranded antiparallel β-sheet. The antiparallel structures are formed at low temperatures in our simulation (Figure 3A and 3B). Do these mutants aggregate through antiparallel β-hairpin structures or by wild-type-like β-helix formation? Thakur and Wetzel's data [5] is compatible with either scenario. If β-hairpin formation is rate-limiting (i.e., nucleates aggregation), since β-hairpin formation by mutants is more thermodynamically favorable than wild type, the aggregation rates of mutants should be higher than wild type. In contrast, if wild type-like β-helix formation in the mutants nucleates aggregation, their aggregation rate compared to the wild type is expected to be determined by the relative stabilities of the metastable mutant and wild-type β-helices. To understand the nucleating conformational transition in these mutant peptides, we performed all-atom MD simulations of a PGQ_9_ sequence in a β-helix conformation (Figure 3C). We found that, similar to the wild type β-helix, the β-helix formed by PGQ_9_ remained stable on the nanosecond time scale, but showed a greater root mean-square distance (RMSD) compared to a wild type β-helix of identical length (see Figure 2A). We compared the RMSD per residue of the wild-type and mutant structures (unpublished data) during MD simulations and found that the destabilization induced by the proline-glycine is not limited to the proline-glycine residues—it is transduced across the whole peptide, leading to an overall higher RMSD. Thus, we propose that the differential stability of the transiently formed β-helix by the mutant peptide compared to wild-type polyQ may underlie the experimentally observed slower rate of aggregation of the mutant.

Mechanisms of protein aggregation [23] are increasingly being sought as a framework for understanding and, importantly, therapeutically interfering with, the fundamental events that underlie misfolding diseases [24]. The common underlying basis of protein aggregation has been demonstrated by the discovery of antibodies can cross-react with early aggregates of different peptides and proteins [25]. Further, the early oligomers themselves, rather than the final fibrils, have been shown to be toxic [26]. Thus, the conversion to specific β-strand topologies is a common central feature associated with cytotoxicity in all aggregation-linked diseases. The structural basis of the mechanism of polyQ peptide aggregation that we present here may thus aid the understanding and development of rational therapies to modulate protein aggregation in these debilitating neurodegenerative diseases.

## Materials and Methods

We modeled the polyQ chain as “beads on a string,” where each glutamine is represented by six pseudo-atoms—four corresponding to the backbone NH, C′, C_α_, and O atoms, and two side chain atoms, one for the methylene (-CH_2_-CH_2_-) groups and another for the carboxylamine (-CONH_2_) group. Neighboring residues in this peptide representation are covalently constrained to mimic the peptide flexibility in real proteins.

To study the conformational dynamics of polyQ, we introduce simplified amino acid interactions: hydrophobic interactions between the methylene groups, polar interactions between sidechains and backbone NH or O beads, and nonspecific backbone hydrogen bonds as described in [16]. The interaction strengths of the hydrophobic interactions, sidechain-backbone polar interactions and nonspecific backbone hydrogen bonds are assigned as 0.7ɛ, 5ɛ, and 5ɛ, respectively. These interaction strengths were used to successfully fold a miniprotein, the Trp-cage, to within 1 Å of its native structure [16]. Interactions in proline and glycine were also modeled as in [16]. We used the rapid DMD algorithm to perform simulations on our model proteins [17,27,28].

We used the snapshots collected from DMD simulations to perform all-atom MD simulations using standard MD protocols. Using a three-step algorithm [29], we reconstructed all atoms of the polyQ chain from the snapshots taken from simulations of coarse-grained protein models. All-atom MD simulations were performed using the package AMBER 7, with the AMBER force-field of parm99 [30,31] at a temperature of 300 Kelvin and pressure of one atmosphere, in a octahedral periodic box of water. The protocol for MD simulations involved equilibration of the solvent and the peptide, and production as described by Urbanc et al. [29]. The trajectory was recorded for 3 ns after equilibration.

Sidechain-backbone interactions have been identified as playing important roles in the formation of protein structures. It has been pointed out that the hydrogen bonds between the polar side chain and backbones are important for the starting and ending of α-helices [32,33] and for the formation of turns in proteins [34]. To evaluate how sensitive our results were to the relative strengths of sidechain and backbone hydrogen bonds, we performed DMD simulations with varying relative strengths of sidechain and backbone hydrogen bonds (ɛ_sidechain_/ɛ_backbone_). We found that for weaker sidechain hydrogen bonds compared to backbone hydrogen bonds, i.e., ɛ_sidechain_/ɛ_backbone_ < 1, polyglutamine (polyQ) formed α-helices at low temperatures as opposed to random coil structures observed at ɛ_sidechain_/ɛ_backbone_ = 1. The observation of random coils at low temperatures is in agreement with experiments in [35], and therefore we chose ɛ_sidechain_/ɛ_backbone_ = 1.

## Abbreviations

DMD: discrete molecular dynamics
MD: molecular dynamics
RMSD: root mean-square distance

## References

[pcbi-0010030-b01] Ross CA, Wood JD, Schilling G, Peters MF, Nucifora FC (1999). Polyglutamine pathogenesis. Philos Trans R Soc Lond B Biol Sci.

[pcbi-0010030-b02] Ross CA, Poirier MA (2004). Protein aggregation and neurodegenerative disease. Nat Rev Neurosci.

[pcbi-0010030-b03] Ross CA (2002). Polyglutamine pathogenesis: Emergence of unifying mechanisms for Huntington's disease and related disorders. Neuron.

[pcbi-0010030-b04] Chen S, Berthelier V, Yang W, Wetzel R (2001). Polyglutamine aggregation behavior in vitro supports a recruitment mechanism of cytotoxicity. J Mol Bol.

[pcbi-0010030-b05] Thakur AK, Wetzel R (2002). Mutational analysis of the structural organization of polyglutamine aggregates. Proc Natl Acad Sci U S A.

[pcbi-0010030-b06] Masino L, Pastore A (2002). Glutarnine repeats: Structural hypotheses and neurodegeneration. Biochem Soc Trans.

[pcbi-0010030-b07] Temussi PA, Masino L, Pastore A (2003). From Alzheimer to Huntington: Why is a structural understanding so difficult?. EMBO J.

[pcbi-0010030-b08] Perutz MF, Finch JT, Berriman J, Lesk A (2002). Amyloid fibers are water-filled nanotubes. Proc Natl Acad Sci U S A.

[pcbi-0010030-b09] Sanchez I, Mahlke C, Yuan JY (2003). Pivotal role of oligomerization in expanded polyglutamine neurodegenerative disorders. Nature.

[pcbi-0010030-b10] Muchowski PJ, Schaffar G, Sittler A, Wanker EE, Hayer-Hartl MK (2000). Hsp70 and Hsp40 chaperones can inhibit self-assembly of polyglutamine proteins into amyloid-like fibrils. Proc Natl Acad Sci U S A.

[pcbi-0010030-b11] Chen SM, Ferrone FA, Wetzel R (2002). Huntington's disease age-of-onset linked to polyglutamine aggregation nucleation. Proc Natl Acad Sci U S A.

[pcbi-0010030-b12] Chen SM, Berthelier V, Hamilton JB, O'Nuallain B, Wetzel R (2002). Amyloid-like features of polyglutamine aggregates and their assembly kinetics. Biochemistry.

[pcbi-0010030-b13] Yang W, Dunlap JR, Andrews RB, Wetzel R (2002). Aggregated polyglutamine peptides delivered to nuclei are toxic to mammalian cells. Hum Mole Genet.

[pcbi-0010030-b14] Scherzinger E, Sittler A, Schweiger K, Heiser V, Lurz R (1999). Self-assembly of polyglutamine-containing huntingtin fragments into amyloid-like fibrils: Implications for Huntington's disease pathology. Proc Natl Acad Sci U S A.

[pcbi-0010030-b15] Altschuler EL, Hud NV, Mazrimas JA, Rupp B (2000). Structure of polyglutamine. FEBS Lett.

[pcbi-0010030-b16] Ding F, Buldyrev SV, Dokholyan NV (2005). Folding Trp-cage to NMR resolution native structure using a coarse-grained protein model. Biophys J.

[pcbi-0010030-b17] Dokholyan NV, Buldyrev SV, Stanley HE, Shakhnovich EI (1998). Discrete molecular dynamics studies of the folding of a protein-like model. Fold Des.

[pcbi-0010030-b18] Marqusee S, Robbins VH, Baldwin RL (1989). Unusually stable helix formation in short alanine-based peptides. Proc Natl Acad Sci U S A.

[pcbi-0010030-b19] Chakrabartty A, Kortemme T, Baldwin RL (1994). Helix propensities of the amino-acids measured in alanine-based peptides without helix-stabilizing side-chain interactions. Protein Sci.

[pcbi-0010030-b20] Blondelle SE, Forood B, Houghten RA, PerezPaya E (1997). Polyalanine-based peptides as models for self-associated beta-pleated-sheet complexes. Biochemistry.

[pcbi-0010030-b21] Wetzel R (2002). Ideas of order for amyloid fibril structure. Structure.

[pcbi-0010030-b22] Stork M, Giese A, Kretzschmar HA, Tavan P (2005). Molecular dynamics simulations indicate a possible role of parallel beta- helices in seeded aggregation of poly-Gln. Biophys J.

[pcbi-0010030-b23] Dobson CM (2003). Protein folding and misfolding. Nature.

[pcbi-0010030-b24] Hammarstrom P, Wiseman RL, Powers ET, Kelly JW (2003). Prevention of transthyretin amyloid disease by changing protein misfolding energetics. Science.

[pcbi-0010030-b25] Kayed R, Head E, Thompson JL, McIntire TM, Milton SC (2003). Common structure of soluble amyloid oligomers implies common mechanism of pathogenesis. Science.

[pcbi-0010030-b26] Kayed R, Sokolov Y, Edmonds B, McIntire TM, Milton SC, at al. (2004). Permeabilization of lipid bilayers is a common conformation-dependent activity of soluble amyloid oligomers in protein misfolding diseases. J Biol Chem.

[pcbi-0010030-b27] Alder BJ, Wainwright TE (1959). Studies in molecular dynamics. I. General method. Journal of Chemical Physics.

[pcbi-0010030-b28] Zhou YQ, Hall CK, Karplus M (1996). First-order disorder-to-order transition in an isolated homopolymer model. Physical Review Letters.

[pcbi-0010030-b29] Urbanc B, Cruz L, Ding F, Sammond D, Khare S (2004). Molecular dynamics simulation of amyloid beta dimer formation. Biophys J.

[pcbi-0010030-b30] Cornell WD, Cieplak P, Bayly CI, Gould IR, Merz KM (1995). A 2nd generation force-field for the simulation of proteins, nucleic-acids, and organic-molecules. J Am Chem Soc.

[pcbi-0010030-b31] Wang JM, Cieplak P, Kollman PA (2000). How well does a restrained electrostatic potential (RESP) model perform in calculating conformational energies of organic and biological molecules?. J Comput Chem.

[pcbi-0010030-b32] Aurora R, Rose GD (1998). Helix capping. Protein Sci.

[pcbi-0010030-b33] Presta LG, Rose GD (1988). Helix signals in proteins. Sci.

[pcbi-0010030-b34] Stickle DF, Presta LG, Dill KA, Rose GD (1992). Hydrogen-bonding in globular-proteins. J Mol Biol.

[pcbi-0010030-b35] Altschuler EL, Hud NV, Mazrimas JA, Rupp B (1997). Random coil conformation for extended polyglutamine stretches in aqueous soluble monomeric peptides. J Pept Res.

